# An Automated Potentiometric System For Precision Measurement Of the Quantized Hall Resistance

**DOI:** 10.6028/jres.092.030

**Published:** 1987-10-01

**Authors:** Giancarlo Marullo Reedtz, Marvin E. Cage

**Affiliations:** Istituto Elettrotecnico Nazionale, Galileo Ferraris, Strada delle Cacce 91, 10135 Torino, Italy; National Bureau of Standards, Gaithersburg, MD 20899

**Keywords:** electrical metrology, ohm, precision measurements, quantized Hall resistance, quantum Hall effect, resistance standards

## Abstract

This paper describes the development of an automated potentiometric measurement system that is used to compare the quantized Hall resistance with that of wire-wound reference resistors having the same nominal value. Conceptual considerations, along with the major practical problems associated with this method, are presented. We then report experimental results which demonstrate that this measurement system is accurate to within a 0.007 ppm one standard deviation uncertainty.

## 1. Introduction

The quantization of the Hall resistance

$$R_{H}(i)=\frac{h}{e^{2}i}\approx \frac{25,812.80}{i}\Omega ,$$ (1)

(where *h* is the Planck constant, *e* the electron charge, and *i* an integer quantum number) has been demonstrated for such diverse two-dimensional electron systems as those in Si-MOSFETs [1]^1^ or in heterostructure devices (GaAs/AlGaAs [2] and more recently InGaAs/InP [3]). This resistance quantization provides a very promising means to yield not only a constant and reproducible as-maintained unit of resistance but to determine also, by solid state physics techniques, the fine structure constant *α* [4,5]. Even if the physical phenomenon (as manifested in real samples) is not completely understood, the experimental results from different national laboratories show an agreement between *R*_H_ values of a few parts in 10^7^ [6] when using different samples and measurement systems.

The precision measurement of *R*_H_ requires three main steps; 1. the metrological characterization of the sample so that a reasonable confidence is reached on the independence of the measured value from the experimental conditions—mainly temperature and current through the sample [7,8]; 2. the comparison of *R*_H_ with the resistance *R*_R_ of a highly stable reference resistor, and 3. the calibration of *R*_R_ in terms of either the as-maintained unit of resistance or in terms of the International System (SI) resistance unit.

An automated potentiometric system has been built to compare, as in step 2, the value of *R*_H_ with a nominally equal reference resistance *R*_R_ within a one standard deviation (1*σ*) uncertainty of 0.007 ppm (parts per million). This system, which replaces and improves an earlier manually-operated version, is flexible enough to be used also for the precision measurements required in step 1.

## 2. Comparison of *R*_H_ and *R*_R_

**Figure 1:**
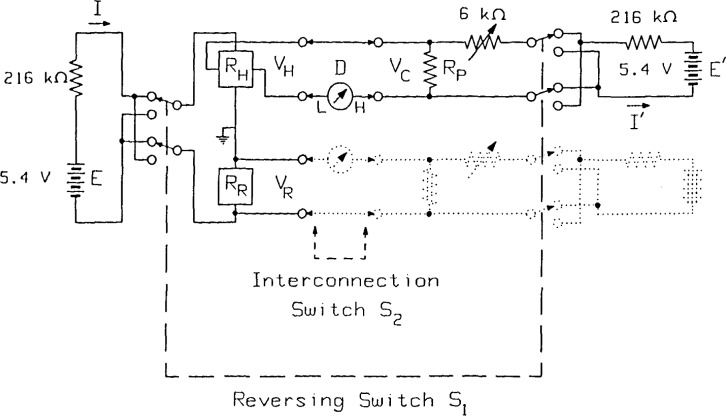
Schematic diagram of the automated potentiometric measurement system which compares the quantized Hall resistance *R*_H_ with a nominally equal reference resistor. The voltage sources *E* and *E*′ are thermally insulated mercury batteries. *D* is a Leeds & Northrup 9829 Linear Amplifier.

The conceptual scheme of the measurement circuit is shown in figure 1, where the numerical values are specific to the case when *R*_H_=6,453.20 Ω (the *i* =4 Hall plateau) and the current is *I* = 25 μA. The two resistances to be compared are connected in series, and the high impedance nanovoltmeter *D* measures the differences between each voltage drop *V*_H_ or *V*_R_ and the compensation voltage *V*_C_. The switch S_1_ enables the current direction and the compensation voltage both to be reversed so that the influence of thermal-electric voltages can be reduced; S_2_ allows for switching between *V*_H_ and *V*_R_. Therefore, S_1_ must not change the resistance of the current circuits too much for the two polarities, and S_2_ must introduce only a minimum amount of thermal voltages in the voltage circuit.

The measurement sequence needed for a single determination of the ratio *R*_H_/*R*_R_ should be chosen to reduce the influence of the thermal voltages and the voltage and current drifts, while also trying to minimize the number of switching operations. Using the letters *H* or *R* to represent the comparisons between *V*_H_ or *V*_R_ and *V*_C_ (switch S_2_), and the symbols + and − for the two current polarities (switch S_1_), we consider the following sequence of operations:

$$R^{+},\;R^{-},\;H^{-},\;H^{+},\;H^{+},\;H^{-},\;R^{-},\;R^{+}.$$ (2)

Within each pair of +/− measurements, the influence of the thermals, if constant, is eliminated by the current reversal. The circuit in figure 1 is also affected by changes in the thermals, and by drifts in the voltage generators *E* and *E*′, or in the currents. Linear drifts in the circuit can be represented as changing currents by the relations

$$\begin{array}{l} I(t)=I(0)(1+\beta t)\\ I^{\prime }(t)=I^{\prime }(0)(1+\beta^{\prime }t). \end{array}$$ (3)

Neglecting constant thermals, we consider a sequence of four measurements *R, H, H, R* where the following relationships hold:

$$\begin{array}{l} I(t_{1})\cdot R_{R}=I^{\prime }(t_{1})\cdot R_{P}-D(t_{1})\\ I(t_{2})\cdot R_{H}=I^{\prime }(t_{2})\cdot R_{P}+D(t_{2})\\ I(t_{3})\cdot R_{H}=I^{\prime }(t_{3})\cdot R_{P}+D(t_{3})\\ I(t_{4})\cdot R_{R}=I^{\prime }(t_{4})\cdot R_{P}-D(t_{4}) \end{array}$$ (4)

where *D*(*t*) is the reading of the detector at time *t*, and *R*_P_ is the potentiometer resistance as labeled in figure 1. If the measurements are uniformly spaced with time intervals Δ*t*, then using eqs (3) and (4) and dividing the sum of the *R*_H_ equations by the sum of the *R*_R_ equations, we obtain

$$\frac{R_{H}}{R_{R}}=\frac{1+\frac{D(t_{2})+D(t_{3})}{2V_{C}(\bar{t})}}{1-\frac{D(t_{1})+D(t_{4})}{2V_{C}(\bar{t})}},$$ (5)

where

$$V_{C}(\bar{t})\equiv R_{P}\cdot I^{\prime }(0)(1+\beta^{\prime }\bar{t})$$ (6)

and $\bar{t}=t_{1}+\frac{3}{2}\Delta t$ is the mean time of the four measurements.

If the two resistance values are different by a small amount we can expand the denominator in eq (5); neglecting second-order terms:

$$\frac{R_{H}}{R_{R}}≅1+\frac{1}{V_{C}}\left[\frac{D(t_{1})+D(t_{4})}{2}+\frac{D(t_{2})+D(t_{3})}{2}\right].$$ (7)

Here the compensation voltage *V*_C_ has been written as time independent, which it is in the limits that we require (e.g., *V*_C_ need only be constant to 1 part in 10^3^ for *D/V*_C_ ratios of 10^−5^ if we want an accuracy of 1 part in 10^8^). Within these limits the drift of *V*_C_ has no influence in eq (7).

We now examine what happens if the switch S_1_ introduces slightly different resistances for the two polarities of the *I* and *I*′ circuits. Let the currents for the two polarities be *I* and (*−I*′ *+* Δ*I*′) for the circuit *E*, and in a similar way *I*′ and (−*I*′ *+* Δ*I*′) for the voltage compensation circuit *E*′. Consider two successive pairs of measurements *R^+^, R*^−^ and *H*^−^, *H^+^*. We can write the relations for the respective detector voltages:

$$\begin{array}{l} D_{R}^{+}=I^{\prime }\cdot R_{P}-I\cdot R_{R}\\ D_{R}^{-}=(-I^{\prime }+\Delta I^{\prime })\cdot R_{P}-(-I+\Delta I)\cdot R_{R}\\ D_{H}^{-}=-(-I^{\prime }+\Delta I^{\prime })\cdot R_{P}+(-I+\Delta I)\cdot R_{H}\\ D_{H}^{+}=-I^{\prime }\cdot R_{P}+I\cdot R_{H}. \end{array}$$ (8)

Subtracting and dividing each pair by two:

$$\begin{array}{l} D_{R}^{*}=\frac{D_{R}^{+}-D_{R}^{-}}{2}=I^{\prime }\cdot R_{P}-I\cdot R_{R}-\frac{\Delta I^{\prime }\cdot R_{P}}{2}+\frac{\Delta I\cdot R_{R}}{2}\\ D_{H}^{*}=\frac{D_{H}^{+}-D_{H}^{-}}{2}=-I^{\prime }\cdot R_{P}+I\cdot R_{H}+\frac{\Delta I^{\prime }\cdot R_{P}}{2}-\frac{\Delta I\cdot R_{H}}{2}. \end{array}$$ (9)

Adding and reordering:

$$\frac{R_{H}}{R_{R}}=1+\frac{D_{R}^{*}+D_{H}^{*}}{I\cdot R_{R}}-\frac{\Delta I}{2I}\left[1-\frac{R_{H}}{R_{R}}\right].$$ (10)

The last (third) term in eq (10) gives a second-order correction if the two resistances to be compared are nearly equal and the variation Δ*I*/*I* is small enough. As before, the denominator of the second term need only be known with limited accuracy and can be replaced by the compensation voltage *V*_C_.

As a conclusion of this analysis we note that: a) the sequence given by eq (2) eliminates the effects of constant thermals within each pair of current reversal measurements; b) the symmetrical sequence eliminates the effects of linear drifts of thermals and of *I* and *I*′; and c) the measurements are not influenced by the slightly different values of *I* and *I*′ for the two current polarities because each polarity is used to compare both *V*_H_ and *V*_R_ with the same compensation voltage *V*_C_.

The measurement sequence actually used is a little more complex but fulfills all the requirements outlined above. It can be represented by

$$\begin{array}{l} R^{+},R^{-},R^{-},R^{+},H^{+},H^{-},H^{-},H^{+},\\ H^{+},H^{-},H^{-},H^{+},R^{+},R^{-},R^{-},R^{+}. \end{array}$$ (11)

This sequence provides a single value of the ratio R_H_/R_R_. If we call the mean values for each group of four measurements *D*_1_, *D*_2_, *D*_3_, and *D*_4_ and if we define

$$\begin{array}{l} D_{R}=\frac{D_{1}+D_{4}}{2}\\ D_{H}=\frac{D_{2}+D_{3}}{2} \end{array}$$ (12)

then the relationship for the *R*_H_*/R*_R_ value is

$$\frac{R_{H}}{R_{R}}=1+\frac{D_{H}}{V_{C}}+\frac{D_{R}}{V_{C}}.$$ (13)

## 3. Sample Characterization and System Flexibility

Every prospective sample must be characterized to establish what experimental conditions are required in order to ensure that the measured values of *R*_H_ have the desired accuracy [7,8]. The following measurements of *R*_H_ and of *R*_X_ (the sample resistance in the direction of the current) are made before a device is considered for precision *R*_H_*/R*_R_ comparisons: 1) R_H_ and *R*_X_ as a function of the sample temperature; 2) *R*_H_ and *R*_X_ as a function of the current through the sample; 3) as in items 1) and 2), but for different pairs of *R*_H_ and *R*_X_ contacts and for both magnetic field directions; and 4) *R*_H_ values for different points along the Hall plateau to verify its flatness.

**Figure 2:**
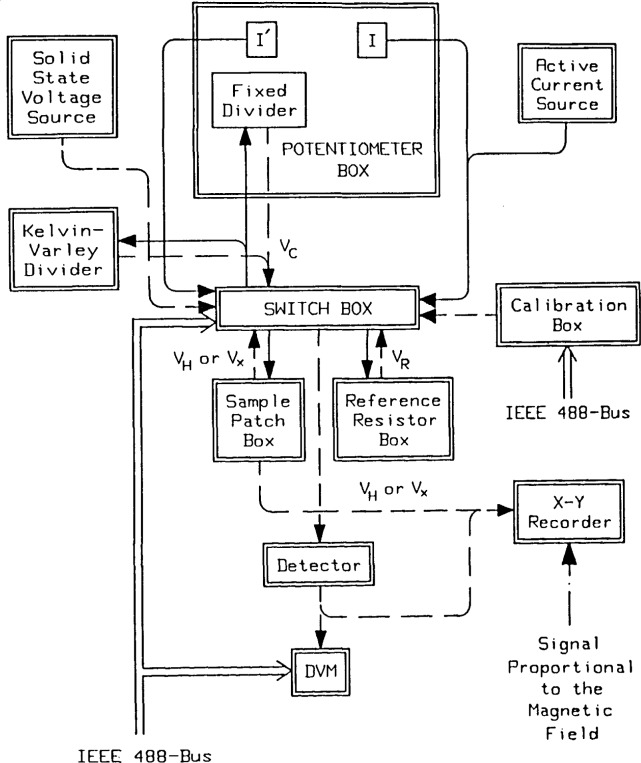
Functional block diagram of the measurement system. This figure stresses the interchangeability of different components to enable the highest precision measurements as well as the physical characterization of the Hall sample. Solid lines represent the current-related functions and dashed lines the voltage-related functions. (There are, of course, actually two paths for every line drawn in the figure.) See the text for further explanation.

All these measurements require precision comparisons between *R*_H_ and *R*_R_. For item 2) the compensation voltage for *R*_H_ measurements must be varied over a wide range. Also, a high impedance current source is required to maintain constant current when the sample is not in a well-quantized regime. The potentiometric comparison circuit of figure 1 has been built in modular form to allow for these different measurement conditions. A functional diagram of the components of this circuit is shown in figure 2, where solid (dashed) lines are for current (voltage) connections. Dashed lines also symbolize output signals that are measured or recorded.

The Potentiometer Box in figure 2 provides the functions needed for the highest precision measurements. The currents *I* and *I*′ are generated using matched sets of thermally insulted mercury batteries and fixed-value resistors. Comparisons of *R*_H_/*R*_R_ can be made at 10 μA and 25 μA for *i* = 4 Hall steps and at 25 μA for *i* = 2 steps. These currents are small enough to provide accuracies better than 1 part in 10^8^ for the three GaAs samples so far characterized.

An electronic, high impedance, constant current source can replace the mercury battery source *I* of the Potentiometer Box for current dependence measurements and for measurements off of the step. The electrical noise, however, is an order of magnitude higher with the constant current source. A high stability solid-state Zener voltage source followed by a Kelvin-Varley resistance divider can substitute for the battery current source and fixed divider. These again are an order of magnitude noisier.

The Sample Patch Box allows for easy connection of the current leads to the source and drain, as well as to any pair of potential pads on the sample, e.g., *V*_H_ or *V*_X_. The voltages read by the detector can either be direct or compensated by *V*_C_.

Each Reference Resistor Box contains an Electro Scientific Industries resistor^2^, composed of series/parallel combinations of wire-wound resistors constructed to have *R*_R_ values within a few ppm of the value of *R*_H_. They are hermetically sealed in a silicone fluid-filled container and placed in a specially constructed temperature-regulated air bath enclosure. The air temperature is controlled to within ±0.002 °C at a nominal temperature of ~28 °C.

The Calibration Box contains an adjustable voltage source whose polarity can be reversed by the Hewlett Packard 9836 computer. This box, when connected to the detector input, enables the detector-digital voltmeter combination to be calibrated.

Finally, *R*_H_ and *R*_R_ can be easily interchanged in the measurement circuit to check for systematic errors. We have found this test to be essential.

## 4. Construction Considerations

### 4.1 The Insulation Resistance

The high values of the resistances to be compared (6,453.20 Ω or 12,906.40 Ω for *i* = 4 or *i* = 2 steps, respectively) require an insulation resistance of the circuit of about 10^12^ Ω to achieve an uncertainty of the order of 10^−8^. As a rule, polytetrafluoroethylene (PTFE or Teflon) has been used as the insulating material. Great care was taken in cleaning the insulating parts of cables and connectors with alcohol. This provides leakage resistances greater than 10^14^ Ω.

The switching is done by Leeds & Northrup 4 pole, 12 position, rotary switches with Lexan wafers (model number 031264). The leakage resistance is 5×10^13^ Ω for each connection. Multiple connections reduce this value. The nanovoltmeter detector D is a Leeds & Northrup 9829 Linear Amplifier. Its input low is only isolated by 10^10^ Ω from the nanovoltmeter guard circuit. Therefore, the detector input low is always connected to the potential point nearest circuit ground, and the guard is also connected to this ground. (The low potential point, of course, changes sides on the sample if the magnetic field direction is reversed.)

The complete potentiomeinc measurement system has an insulation resistance of 3×10^12^ Ω when the humidity is less than 50%. This leakage resistance is periodically monitored to ensure that dust, lint, hair, and fingerprints have not reduced it.

### 4.2 Contact Resistances, Thermal-Electric Voltages, and Drifts

The cables consist of sets of twisted pairs of PTFE-insulated, silvered, stranded copper wires enclosed within two separate coaxial shields. British Post Office connectors (model numbers OFF 66148 and 66149) are used whenever possible because they have small and reproducible contact resistances, high leakage resistances, and are completely shielded.

Fixed connections are made by a low melting point Sn-Pb alloy solder, rather than a low thermoelectric power Sn-Cd alloy, because of better mechanical strength and more reliable electrical contacts. Tightly-packed thermal insulation is used to minimize temperature gradients.

The contact resistances of the Leeds & Northrup rotary switches are less than 0.7 mΩ and have a reproducibility of 0.05 mΩ. Differences in resistance of the two current circuits, due to slightly different lengths of the wires through the switches for each polarity, are less than 4 mΩ. That value is less than 2×10^−8^ of the resistance of each circuit. As shown in section 2, this has no detectable influence on the result when using the sequence of measurements shown in expression (11). These switches exhibit less than 20 nV thermals 10 seconds after repeated switching.

The drifts of the *I* and *I*′ sources are of the order of 0.2 ppm or less during the 12 minute time intervals required to carry out the measurement sequence of expression (11). This small drift rate is achieved by using careful thermal insulation of the Potentiometer Box, and by not opening the loads during current reversals. (Momentary double loads in the make-before-break switching are better than open-circuiting for the stability of the mercury batteries.)

### 4.3 The Automated Switching

Stepping motors have been used to rotate the switches S_1_ and S_2_. They are Clifton Precision model 23-SHAB-49BU motors with a minimum rotation of 0.9°/step and a 0.45 N·m torque. The motors must overcome a significant frictional force. As a result, they occasionally can miss steps. After several misses the switches may open-circuit. To prevent this we realign the switches by deliberately forcing the motors to rotate the switches against stops.

The motors are controlled by a driver (a Clifton Precision model DPB05) which supplies the clock pulses that control the steps, and other TTL signals which determine the direction of rotation, the working mode (0.9°/step or 1.8°/step), and the current-off-after-switching capability. The last feature is useful to reduce the noise in the system during measurements and to allow for manual switch operation.

### 4.4 Noise Problems

Great care has been used in shielding the system. Two independent levels of electrostatic shielding have been provided for all the boxes and cables within the instrument. In addition, the electronics rack for the instrument, aluminum shelves for the reference resistors, metal room walls, and copper pipe for the signal cable to the cryostat give a third, nearly complete, shielding level that is electrically insulated from the other two shields. The only exceptions are: the reference resistor boxes which have only two levels of shielding, and the cryostat and quantum Hall sample holder with just one independent shield.

To avoid current loops, all cables have their coaxial shields broken on one side. Shielding connections among different chassis boxes are made by dedicated cables. Only one connection exists between different shielding levels, between the instrument rack and laboratory ground, and between the first shield and the measurement circuit. This last connection can be opened for the leakage resistance tests.

The largest amount of electrical noise in the system comes from the nanovoltmeter detector, which has 150 nV peak-to-peak noise within a 0.5 Hz bandwidth. This noise (obtained with the input short-circuited and with the instrument powered by batteries) corresponds to about 38 nV at the 1*σ* level, to be compared with 7 nV (1*σ*) Johnson noise of a 6,453.20 Ω resistor at room temperature within the same bandwidth.

The nanovolt amplifier noise is about 25×10^−8^ of the voltage to be compared for a current of 25 μA on the *i* =4 step. Therefore, long integration times must be used on the detector output to obtain uncertainties less than 1×10^−8^ for the standard deviation of the mean of a *R*_H_*/R*_R_ comparison.

For each operation of the sequence given in expression (11), the computer takes 60 readings in 30 s time intervals from a digital voltmeter at the output of the detector. A wait time of 15 s is introduced after switching. It takes 12 minutes to complete one sequence and thus yield a single value of the ratio *R*_H_/*R*_R_. The standard deviation^3^ of such a sequence is typically 0.016 ppm. The value is the same whether the reference resistor is compared with a quantum Hall resistor or with another reference resistor. Seven sequences (84 minutes of measurements) are usually sufficient to obtain a standard deviation of the mean (random uncertainty) of 0.006 ppm.

## 5. System Performance

This measurement system has been in operation since August, 1986 to compare the value of *R*_H_ for the *i* = 4 plateau of a GaAs/AlGaAs heterostructure with that of two nominally equal 6,453.20 Ω reference resistors, and to also intercompare the two reference resistors. Table 1 summarizes the estimated uncertainties assigned to quantum Hall sample-reference resistor intercomparisons for a 25 μA current.

The standard deviation of the mean (the random or type A measurement uncertainty) is, as noted above, typically ±0.006 ppm for a sample-resistor comparison. The position of the sample and resistor are then interchanged in the measurement system and the values are remeasured. The Hall resistance value assigned is the unweighted mean of these two comparisons, and the random uncertainty of the 14 pooled measurements is usually ±0.004 ppm. Pooling all of the measurements is viewed as justified since usually the difference in the values obtained in the two positions is well within the scatter of the data.

In addition to the random uncertainties, there are systematic, or type B, uncertainties associated with systematic corrections and other effects. One such correction is due to a measurement system offset, or interchange, error in which the value of the Hall resistance depends on whether it is measured in the *R*_H_ position or in the *R*_R_ position of the measurement circuit. The largest interchange correction to date has been ±0.013 ppm, but, as noted above, is usually much less (i.e., indiscernible; the correction is based on the assumption that the mean of the two values obtained is the correct value). It is not completely understood why the interchange correction is less of a problem in this measurement system than it is in the old version of the potentiometric system [9] and in the automated bridge system [9,10], Perhaps it is due to a higher leakage resistance and to better electrical shielding. The estimated type B uncertainty associated with the interchange correction is assumed to be the same as the random uncertainty: ±0.004 ppm.

There is an uncertainty in calibrating the gain of the electronic detector-digital voltmeter pair. The pair can *appear* to vary by a few tenths of a percent over the input voltage range if the DVM has a dead-band at zero volts [9]. The dead-band of our Hewlett Packard 3457A Digital Multimeter is small enough for this effect to be negligible. There remains, however, the problem of the *stability* of the detector-digital voltmeter gain. The day-to-day gain varies by ~0.1% when the room temperature is controlled to ± 1 °C. This instability contributes an estimated ±0.003 ppm type B uncertainty to the sample-resistor measurements because our reference resistors are about 2–3 ppm smaller than the quantum Hall resistance.

The 3×10^12^ Ω measurement system leakage resistance contributes an estimated ±0.002 ppm type B uncertainty.

No temperature-dependent [7] or current-dependent [8] quantized Hall resistance (QHR) corrections could be detected for the Hall-probe set used on this sample. The respective estimated type B uncertainties for the dependence of the QHR on temperature and current are ±0.002 ppm and ±0.001 ppm.

The total root-sum-square uncertainty is typically ±0.007 ppm. This uncertainty is only one third that of the old potentiometric measurement system [9] and the bridge system [9,10]. The smaller uncertainty owes to: a) the ability to directly interchange the sample and the reference resistor positions in the measurement system, rather than having to substitute another reference resistor for the sample in order to determine the interchange error; b) a smaller interchange error; c) the fact that there is only one uncorrelated uncertainty of the detector-digital voltmeter gain, rather than the three correlated uncertainties in the bridge system; and d) a larger leakage resistance.

## 6. Results

**Figure 3:**
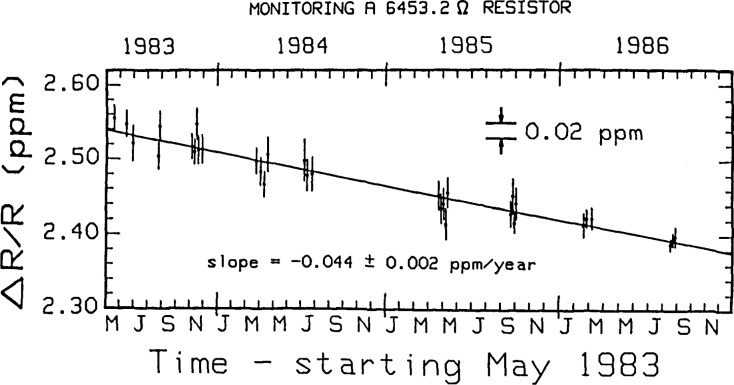
Relative comparisons as a function of time of the resistance of the *i* =4 steps of three different quantum Hall devices with that of a nominal 6,453.20 Ω wire-wound reference resistor. Δ*R/R* =(*V*_H_ − *V*_R_*)/V*_R_. The value of this resistor is increasing by (0.044±0.002) ppm/year. The last set of data points were obtained with the new measurement system.

The United States legal unit of resistance Ω_NBS_ is being monitored via the quantum Hall effect (9), as outlined in steps 1 to 3 of section 1. Comparisons between *R*_H_ and *R*_R_ are made as part of this ongoing procedure. Figure 3 displays the current results. The value of the 6,453.20 Ω reference resistor used in the comparisons is increasing by (0.044 ±0.002 ppm/year). The August 1986 data shows the improved measurement accuracy of the new automated potentiometric system over that of the old, manually-operated potentiometric measurement system and the automated bridge system. This new measurement system has proved to be quite reliable, very flexible, and easy to use.

## Figures and Tables

**Table 1:** Estimated one standard deviation (68% confidence level) uncertainties for the quantum Hall resistance measurements at 25 μA.

*R*_H_↔*R_R_*	

Sources of Uncertainty	Uncertainty (ppm)
Random	0.004
Sample-Resistor Interchange	0.004
Detector Gain Stability	0.003
Leakage Resistance	0.002
QHR Temperature Dependence	0.002
QHR Current Dependence	0.001
ROOT-SUM-SQUARE TOTAL (ppm)	0.007

## Footnotes

1 Figures in brackets indicate literature references.

2 Brand names are used only for purposes of identification. Such use implies neither endorsement by the National Bureau of Standards nor assurance that the equipment is the best available.

3 Throughout, all uncertainties are meant to correspond to one standard deviation estimates.
